# Dorsal periaqueductal gray simultaneously modulates ventral subiculum induced-plasticity in the basolateral amygdala and the nucleus accumbens

**DOI:** 10.3389/fnbeh.2015.00053

**Published:** 2015-03-04

**Authors:** Omer Horovitz, Gal Richter-Levin

**Affiliations:** ^1^The Institute for the Study of Affective Neuroscience (ISAN), University of HaifaHaifa, Israel; ^2^Department of Psychology, University of HaifaHaifa, Israel; ^3^Sagol Department of Neurobiology, University of HaifaHaifa, Israel

**Keywords:** affect, plasticity, modulation, BLA, NAcc, dPAG

## Abstract

The ventral subiculum of the hippocampus projects both to the basolateral amygdala (BLA), which is typically, associated with a response to aversive stimuli, as well as to the nucleus accumbens (NAcc), which is typically associated with a response to appetitive stimuli. Traditionally, studies of the responses to emotional events focus on either negative or positive affect-related processes, however, emotional experiences often affect both. The ability of high-level processing brain regions (e.g., medial prefrontal cortex) to modulate the balance between negative and positive affect-related regions was examined extensively. In contrast, the ability of low-level processing areas (e.g., periaqueductal gray—PAG) to do so, has not been sufficiently studied. To address whether midbrain structures have the ability to modulate limbic regions, we first examined the ventral subiculum stimulation’s (vSub) ability to induce plasticity in the BLA and NAcc simultaneously in rats. Further, dorsal PAG (dPAG) priming ability to differentially modulate vSub stimulation induced plasticity in the BLA and the NAcc was subsequently examined. vSub stimulation resulted in plasticity in both the BLA and the NAcc simultaneously. Moreover, depending on stimulus intensity, differential dPAG priming effects on LTP in these two regions were observed. The results demonstrate that negative and positive affect-related processes may be simultaneously modulated. Furthermore, under some conditions lower-level processing areas, such as the dPAG, may differentially modulate plasticity in these regions and thus affect the long-term emotional outcome of the experience.

## Introduction

A core aspect of *affect* is the integral blend of hedonic (negative-positive/displeasure-pleasure) values (Russell, 2003). In accordance with that, evidence from pre-clinical studies suggests the involvement of both *negative* and *positive* brain affect-related systems in emotional experiences (Panksepp, 1998). Traditionally, neurobiological studies of emotions focus on either *negative* or *positive* affect system. However, these affective systems are likely to function simultaneously to form a functional balance between them. The hippocampus is interconnected with regions that are typically associated with affective valence. For example, the Ventral Subiculum (vSub) projects to the Basolateral Amygdala (BLA; Maren and Fanselow, 1995; Davis, 1997), as well as to the Nucleus Accumbens (NAcc; Brog et al., 1993). The BLA is strongly associated with responses to aversive stimuli (LeDoux, 1993, 1996, 2003, 2007; Ledoux and Muller, 1997; Emery and Amaral, 2000; Davis and Whalen, 2001; Cardinal et al., 2002; Balleine and Killcross, 2006; Shin et al., 2006) while the NAcc is strongly associated with positive-appetitive stimuli (for a review see: van Praag et al., 2000). Morrison and Salzman (2010) proposed that the amygdala can act as a general appetitive (*positive*)-aversive (*negative*)-affective module which is thought to be involved also in reward-based decision-making tasks. Likewise, Cardinal et al. (2002) proposed that the NAcc can play a bivalent role in the modulation of goal direct actions that are affected by either safety or danger cues experienced in the environment. Projections of the BLA into the NAcc which are involved in the modulation of cue-triggered motivation behaviors (Stuber et al., 2011), further strengthen this claim.

Modulation of these regions is mainly attributed to “higher-level” processing structures such as the medial prefrontal cortex (mPFC, for example see: Amygdala—Marek et al., 2013; NAcc—Richard and Berridge, 2013). Far less is known about modulation by “lower-level” processing structures such as those located in the midbrain. For example, contemporary fear-conditioning models present the midbrain Periaqueductal Gray (PAG) as downstream of the amygdala (Da Costa Gomez et al., 1996). However, in a previous study we have shown that electrically priming dPAG resulted in modulation of plasticity in subiculum–BLA synapses, providing evidence also for upstream modulation of the amygdala by the dPAG (Kim et al., 2013). In the current study, we extended these findings to test whether dPAG priming can simultaneously modulate plasticity in both the BLA and the NAcc. Furthermore, we examined whether under specific conditions, dPAG could have differential impact on BLA and NAcc plasticity. Since previous studies proposed that moderate threatening stimuli inhibit the dPAG and that this inhibition may be overcome with more extreme danger (Deakin and Graeff, 1991; Graeff et al., 1993), the modulation of vSub-induced plasticity in the BLA and NAcc under different dPAG priming stimulation intensities was also examined.

## Materials and methods

### Experimental animals

40 male Sprauge-Dawley rats supplied by Harlan Laboratories Jerusalem at the ages of 50 days were used.

### Housing conditions

Rats were grouped housed in plastic storage cages (35 × 60 × 18 cm) on sawdust bedding. The laboratory vivarium maintains an automatic 12 h light-dark cycle (on at 7:00 am). The sawdust bedding was replaced once a week. Water and food, (Teklad Global Diet 20185, Harlan Teklad Ltd., WI, USA) *ad libitum*. All experimental procedures and assessments were performed in designated rooms away from the vivarium, during the light phase, adhered to the NIH Guide for the care and use of laboratory animals and were approved by the University of Haifa ethical committee.

### Experimental groups

Rats were randomly assigned to one of four experimental groups:

1. vSub HFS group: recording simultaneously from the BLA and NAcc following vSub HFS (*n* = 12).
2. dPAG HFS group: recording simultaneously from the BLA and NAcc following dPAG HFS (*n* = 9).
3. dPAG priming 0.5 mA + vSub HFS group: recording simultaneously from the BLA and NAcc following dPAG priming at 0.5 mA of vSub HFS (*n* = 9).
4. dPAG priming 1.0 mA + vSub HFS group: recording simultaneously from the BLA and NAcc following dPAG priming at 1.0 mA of vSub HFS (*n* = 10).

### Experimental design

Following delivery (PND 50), animals were housed in the laboratory vivarium for 5 days of acclimation. Starting on the 6th day, 2 animals were anesthetized per day and underwent electrophysiological assessments. Immediately following the electrophysiological assessment, animals were decapitated and their brains were harvested for electrodes positioning by histology (i.e., Cresyl Violet staining).

### Surgical procedure

In preparation for electrophysiological recording, rats were anesthetized with an intraperitoneal injection (IP) of Urethane (0.5 mg/1 kg body weight) and mounted in a stereotaxic apparatus (Stoelting Co. Illinois, USA). The scalp was incised and retracted, and head position was adjusted to place Bregma and Lambda in the same horizontal plane. Small burr holes (2 mm diameter) were drilled unilaterally in the skull for the placement of stimulating and recording electrodes (described below). A 125 µm coated wire reference electrode was affixed to the skull in the area overlapping the nasal sinus on the contra-lateral side. Placement of the stimulating electrode was done according to the stereotaxic criteria and based on preliminary experiments. Stimulating electrodes were implemented in the dPAG and the vSub. Using stereotaxic criteria, auditory signals generated from multiple-unit discharges and according to preliminary experiment designed specifically to validate regions coordinates; the recording electrodes were implemented in the BLA and NAcc. During the course of experiments, body temperature was maintained at 36.5–37.4°C with a feedback regulated temperature controller (FHC, Bowdoinham, ME, USA).

### Electrodes positioning

Stimulation electrode was positioned in the vSub (AP: −6.3 mm; ML: 5 mm; DV: ((−6)—(−8)mm)). For priming the dPAG, additional stimulating electrode was positioned in the dPAG (AP: −6.05; ML: 0.64; DV: −5.72). Recording electrodes were positioned in the BLA (AP: −3.2 mm; ML: 5 mm; DV: ((−7)—(−7.5) mm)) and the NAcc (AP: 1.6 mm; ML: 0.9 mm; DV: ((−5.5)—(−6.4) mm)).

### Electrodes characteristics

Bipolar concentric stimulating electrodes (125 µm; Kopf, Tujunga, CA) were used for the stimulation of the vSub and the dPAG. For recording in the BLA and the NAcc we have used stainless steel recording electrodes (tip diameter, 2 µm; 20 mm length; Plastic One Inc., model: E363/2/SPC ELEC 0.008-SS).

### Electrophysiological recording

For each rat, measurements of input-output curve responses (0.2 mA up to 1.8 mA) were conducted to determine baseline stimulation intensity. A 20 min pre-HFS baseline was collected at stimulation intensity that reached 35–40% of the maximum peak height (PH) response collected during input-output recordings in both the BLA and the NAcc. For testing vSub ability to induce plasticity in BLA and NAcc, High Frequency Stimulation (HFS) train consisting of stimulating (the vSub) for 10 brief bursts (200 ms) of 100 Hz stimulation delivered at 1 Hz (a total of 200 pulses) was used. Rats received 4 HFS trains separated by 5 min (i.e., ISI). Responses were collected (once every 20 s) during baseline session and for 60 min following the last stimulation session (partially adapted from Maren and Fanselow, 1995). For testing dPAG priming on BLA and NAcc plasticity following vSub HFS, priming stimulation was composed of a single HFS train to the dPAG delivered in one of two intensities: 1.0 mA or 0.5 mA 10 s before the application of HFS to the vSub as described above.

### Calculating ratio peak height

**Figure 1:**
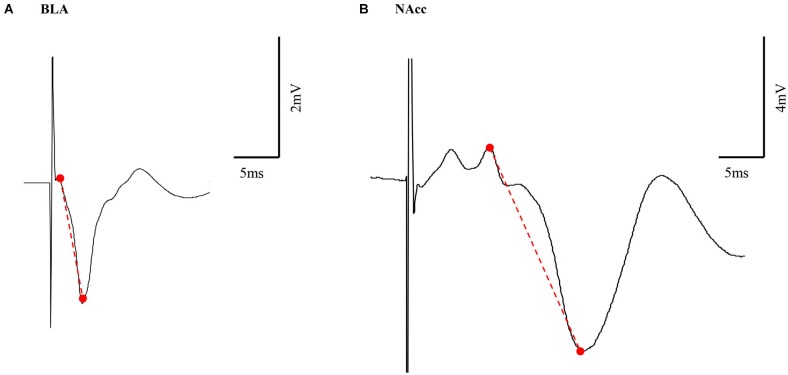
**Representative example of BLA and NAcc field potential responses**. Peak height (PH) amplitude was measured from the highest peak before a trough to the lowest peak (marked in a red dashed line) in both the BLA **(A)** and NAcc **(B)**.

In both the BLA and the NAcc, the principal measure of size of the averaged evoked field potentials was peak-to-peak amplitude. Peak height (PH—here after) amplitude is defined from the highest peak before a trough to the lowest peak (Figure 1).

#### Histology

At the completion of the electrophysiological assessment, animals were overdosed with Ureathane. Their brains were removed and frozen in powdered dry ice and stored at −80°C until sectioning. Coronal sections (40 µm) along the extent of the electrodes lesions were cut using a cryostat (Leica Microsystems Inc.) at −20°C, mounted on gelatin-coated slides, and stained with cresyl violet.

#### Statistical analyses

All statistics were conducted by using ANOVA with repeated measures in SPSS 20. All *post hoc* tests were conducted by using Bonferroni comparisons.

## Results

### Body weight

All groups were weighted prior to the electrophysiological assessments. One-way ANOVA for averaged weights of the different groups did not reveal any significant effects (*F*_(3,36)_ = 0.358, n.s.).

### Simultaneously induced plasticity in BLA and NAcc and the effects of dPAG priming Plasticity in BLA and NAcc induced by electrical stimulation to the vSub

**Figure 2:**
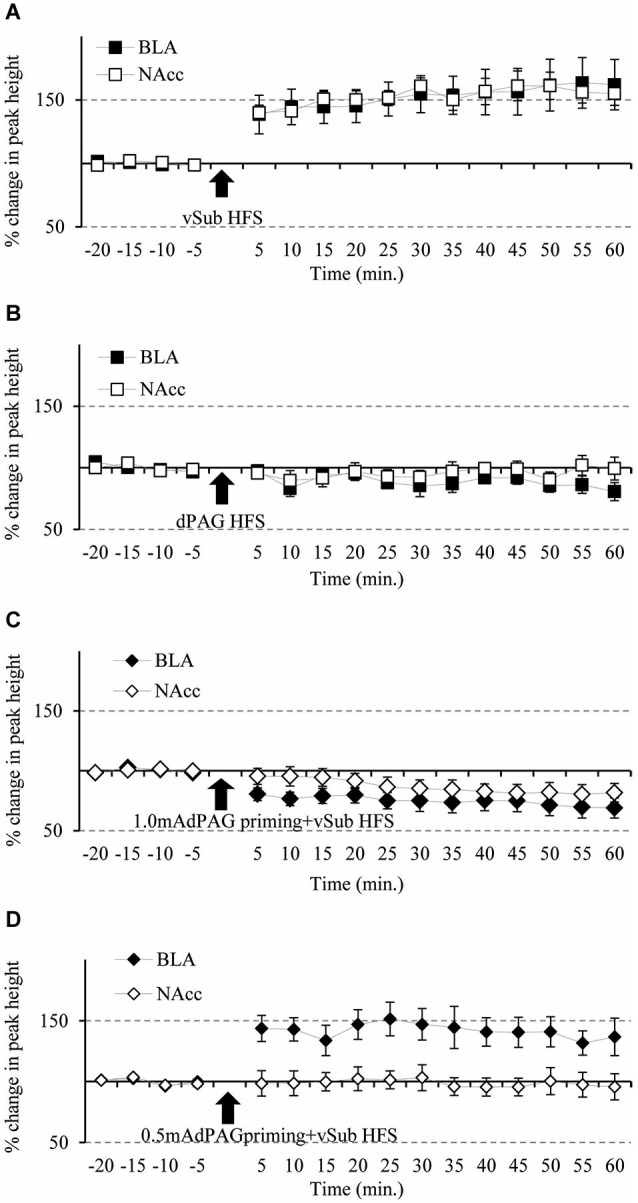
**Simultaneous plasticity and meta-plasticity in the BLA and the NAcc. (A)** The effects of vSub HFS on BLA and NAcc plasticity. Compared to baseline recordings, vSub HFS significantly induced plasticity in the form of LTP in both the BLA and the NAcc simultaneously, (*n* = 12; *p* = 0.05). **(B)** The effects of dPAG HFS on BLA and NAcc plasticity. Compared to baseline recordings, dPAG HFS failed to induce any significant plasticity in both the BLA and the NAcc simultaneously, (*n* = 9; *n.s.*). **(C)** The effects of vSub HFS + 1.0 mA dPAG priming on BLA and NAcc plasticity. Compared to baseline recordings, vSub HFS + 1.0 mA dPAG priming induced significant plasticity in the form of LTD in both the BLA and the NAcc simultaneously, (*n* = 10; *p* = 0.05). **(D)** The effects of vSub HFS + 0.5 mA dPAG priming on BLA and NAcc plasticity. Compared to baseline recordings, vSub HFS + 0.5 mA dPAG priming induced significant plasticity in the form of LTP in the BLA whereas no significant plasticity was observed in the NAcc, while recording simultaneously in both regions, (*n* = 9; *p* = 0.05—for BLA only).

*In vivo* electrophysiology measurements were performed to test for synaptic plasticity in both the BLA and NAcc pathways following HFS to the vSub. Comparisons between time points using ANOVA with repeated measures before the application of HFS did not revealed any significant difference in PH amplitudes (*F*_(3,36)_ = 0.876, n.s.). Following HFS, repeated measure ANOVA revealed a significant effect for the different stimulation types on the ability to induce plasticity (*F*_(3,36)_ = 23.37, *p* = 0.000). Figures 2A–D present *post hoc* comparisons related to plasticity in the BLA and the NAcc (*p* = 0.05). vSub HFS induced simultaneous LTP in the BLA and the NAcc (Figure 2A). dPAG stimulation by itself did not have significant effects on the responses in the BLA or in the NAcc (Figure 2B). In contrast, 1.0 mA dPAG priming that preceded HFS to the vSub resulted in simultaneous LTD in the BLA and the NAcc (Figure 2C). However, 0.5 mA dPAG priming that preceded the vSub HFS did not block LTP in the BLA while it did so in the NAcc (Figure 2D).

**Figure 3:**
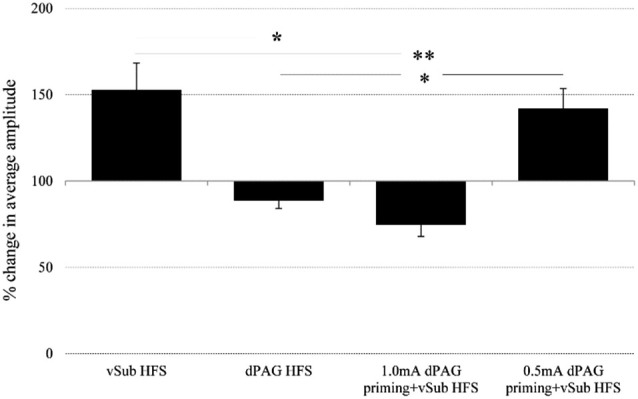
**Averaged plasticity in the BLA**. In the BLA, average 60 min. post baseline plasticity revealed that vSub HFS (*n* = 12) LTP differed from dPAG HFS (*n* = 9) and 1.0 mA dPAG priming + vSub HFS (*n* = 10) LTD. 0.5 mA dPAG priming + vSub HFS (*n* = 9) resulted in LTP which differed from dPAG HFS and 1.0 mA dPAG priming + vSub HFS which resulted in LTD. (*< 0.05, **< 0.001).

Within the BLA, ANOVA for repeated measures for time points during baseline recordings did not reveal any significant difference between the different groups (for averaged amplitudes (Mean ± SEM) please see, Table 1; *F*_(3,36)_ = 1.04, n.s.). However, significant differences between groups were found following the application of HFS (*F*_(3,36)_ = 11.19, *p* = 0.000). Figure 3 depicts significant differences revealed by *post hoc* Bonferroni comparisons between the groups. vSub HFS group (*n* = 12) which demonstrated significant LTP (detailed in Figure 2A), differed significantly from the dPAG HFS group (*n* = 9) that exhibited no change from baseline (detailed in Figure 2B) (*p* = 0.002). A significant difference was found also between the vSub HFS group (detailed in Figure 2A) and the 1.0 mA dPAG priming + vSub HFS group (*n* = 10), which exhibited LTD instead of LTP (detailed in Figure 2C) (*p* = 0.000). In addition, a significant difference was found between the 0.5 mA dPAG priming + vSub HFS group (*n* = 9) and the 1.0 mA dPAG priming + vSub HFS group (*p* = 0.002). While 0.5 mA dPAG priming + vSub HFS resulted in a form of LTP (detailed in Figure 2D), priming dPAG at 1.0 mA resulted in a form of LTD (detailed in Figure 2C), as indicated by a significant reduction in the BLA response of that group compared with the vSub HFS group (detailed in Figure 2A) (*p* = 0.026).

**Table 1:** **Averaged baseline peak height amplitudes (mV)**.

Groups	BLA	NAcc
vSub HFS (*n* = 12)	2.83 ± 2.18	1.92 ± 2.45
dPAG HFS (*n* = 9)	1.45 ± 1.17	2.70 ± 1.48
1.0 mA dPAG priming + vSub HFS (*n* = 9)	2.42 ± 2.37	3.02 ± 1.29
0.5 mA dPAG priming + vSub HFS (*n* = 10)	2.54 ± 1.43	3.64 ± 0.60

*Averaged peak height amplitudes of the different experimental groups in the BLA and the NAcc, collected during 20 min. baseline recordings*.

**Figure 4:**
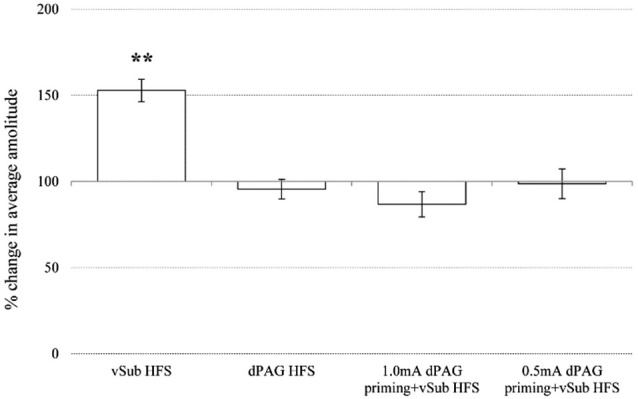
**Averaged plasticity in the NAcc**. In the NAcc, average 60 min post baseline plasticity revealed that vSub HFS (*n* = 12) LTP differed from dPAG HFS (*n* = 9), 1.0 mA dPAG priming + vSub HFS (*n* = 10) LTD, and 0.5 mA dPAG priming + vSub HFS (*n* = 9) (** < 0.001).

Within the NAcc, ANOVA for repeated measures for time points during baseline recordings did not reveal any significant differences between the different groups (Table 1; *F*_(3,36)_ = 2.05, n.s.). After applying priming stimulation and HFS protocols, however, significant group differences were observed (*F*_(3,36)_ = 20.23, *p* = 0.000). Figure 4 depicts significant differences revealed by *post hoc* Bonferroni comparisons between the groups. vSub HFS group (*n* = 12) significantly differed from all other groups (i.e., dPAG HFS (*n* = 9, *p* = 0.000), 0.5 mA dPAG priming + vSub HFS (*n* = 9, *p* = 0.000), and 1.0 mA dPAG priming + vSub HFS (*n* = 10, *p* = 0.000)). While vSub HFS resulted in a form of LTP (detailed in Figure 2A), priming dPAG at 0.5mA + vSub HFS prevented LTP induction (detailed in Figure 2D), and priming dPAG at 1.0 mA + vSub HFS resulted in a form of LTD (detailed in Figure 2C), as indicated by a significant reduction in the NAcc response of that group compared with the vSub HFS group and the 0.5 mA priming + vSub HFS group (detailed in Figures 2A,D) (*p* = 0.000).

## Discussion

The current study was designed to explore plasticity in affect-related brain structures, in a network consisting of higher limbic regions and the limbic midbrain. Independent indications have previously shown that the vSub projects to the BLA (Maren and Fanselow, 1995; Kim et al., 2013) or to the NAcc (Aylward and Totterdell, 1993; Lisman and Grace, 2005); our data demonstrates the possibility for simultaneously inducing plasticity in both regions. HFS stimulation to the vSub induced plasticity in the form of LTP in both the BLA and NAcc at the same time.

Although most investigations of amygdala functions focus on aversive learning, it was also suggested that the BLA might be particularly important for maintaining and updating the representation of the affective value of appetitive stimuli (Parkinson et al., 1999, 2001). On the other hand, Hikida et al. (2013) suggested that reward and aversive learning are regulated by pathway-specific neural plasticity in the NAcc. This is in line with other studies that pointed on the NAcc role in various tasks involving aversive motivation (for reviews, see: Salamone, 1994; Pezze and Feldon, 2004). Indeed, it was shown that amygdala–striatal interactions are critical for processing of information about learned motivational value (Setlow et al., 2002). Taken together, Delgado et al. (2008) suggested that understanding the relationship between aversive and appetitive reinforcement and the processes underlying the complementary roles of the amygdala and striatum would become increasingly important in the development of comprehensive models. The ability of simultaneously assessing activity, plasticity and the balance between these two regions presents a useful tool for verifying these possibilities, as suggested by Delgado et al.

Anatomically, Maren and Fanselow (1995) have previously noted that a prominent source of BLA afferents is the hippocampus, specifically by projections arisen from the vSub. It is also known that these vSub-BLA projections are monosynaptic (Mello et al., 1992) and that stimulating the first can modulate BLA field potentials synaptic transmission (Maren and Fanselow, 1995; Kim et al., 2013). It should be noted though that others have pointed out that in fact only a minor part of the ventral subiculum afferents innervates specifically the BLA (for example: Canteras and Swanson, 1992) and therefore, intra- amygdalar pathways should also be taken into consideration when addressing vSub-BLA plasticity as we did here. It is nevertheless interesting to note that despite the proposed restricted nature of vSub innervation of the BLA, the dPAG was able to modulate activity in the BLA that resulted from vSub activation, indicating the robustness of the dPAG modulation of BLA activity. Additionally, it was shown that the subiculum is connected to the NAcc (Groenewegen et al., 1987). LTP in the NAcc by stimulating the projections from the hippocampal formation was first described by Boeijinga et al. (1990) and was confirmed by others since then (Mulder et al., 1997, 1998; Dong et al., 2007). Finally, an amygdala-NAcc interaction was also documented (Aylward and Totterdell, 1993).

Functionally, the vSub is a principal route by which the hippocampus communicates with the amygdala to activate a contextual fear system (Biedenkapp and Rudy, 2009). It was found that damage to the vSub after training in a contextual conditioning produced a major impairment, although a lesion prior to conditioning had an insignificant effect (Maren, 1999; Biedenkapp and Rudy, 2009). It was also demonstrated that increasing plasticity potential in the BLA (by injecting a D1 agonist) could compensate for the lack of vSub input. These results indicate that under normal conditions the vSub-BLA pathway is critical for contextual fear conditioning to be manifested, but that under some conditions the contribution of the vSub-BLA input could be replaced by enhanced plasticity within the BLA (Biedenkapp and Rudy, 2009). These and other results (Guarraci et al., 1999) are in line with the hypothesis that there are two neural systems that can support contextual fear conditioning—one that contains the hippocampal formation and one that relies on subcortical activation. While under normal conditions the former is the dominant system under yet-to-be defined conditions the other system may take over, probably altering the nature of the contextual memory that is formed (Kogan and Richter-Levin, 2008, 2010). It is tempting to suggest that activation of the dPAG may serve to shift the relative dominance of such response systems. The vSub is also suggested to act as an interface between the hippocampus as a contextual information processor and subcortical processing systems related to motivation, sensory integration and motor output, such as the ventral striatum (Quintero et al., 2011). Considerable evidence suggests that the NAcc has an important role in hippocampus-dependent processing of spatial and contextual cues (Annett et al., 1989; Riedel et al., 1997; Setlow and McGaugh, 1999), in BLA-mediated reward conditioning (Cador et al., 1989; Everitt et al., 1991) and in discrete cue fear conditioning (Pezze et al., 2002). Although not studied yet in a similar way, it is reasonable to assume that modulation of the dPAG could alter the relative dominance of subcortical vs. hippocampal influences over these behaviors. In our study, after establishing the ability to simultaneously induce plasticity in the vSub to the BLA and NAcc pathways, limbic midbrain modulation of this plasticity was examined by priming of the dPAG. The importance of limbic modulation on midbrain structures such as the PAG is well established (e.g., Adamec, 1997, 1998, 2001). With respect to that, Behbehani (1995) described the PAG as a major site for processing of fear and anxiety. It also interacts with the hippocampus formation. For example, Temel et al. (2012) reported that electrical stimulation of the dlPAG increased the number of c-Fos immunoreactive cells in specific sub-regions of the hippocampus. PAG also interacts with the amygdala (Behbehani, 1995). Since stimulation of either the amygdala or the dPAG produces fear and anxiety (Graeff et al., 1993), this Amygdala-PAG pathway is believed to play a crucial role in the fear system (Da Costa Gomez et al., 1996). For example, it was previously shown that Amygdala- PAG relations are important in modulating anxious-like behavior in both cats (Adamec, 1997) and rats (Adamec, 2001). De Oca et al. (1998) suggested that dorsolateral PAG (dlPAG) might inhibit the amygdala or other forebrain structures involved in processing fear-provoking stimuli. Finally, there are several reports which support the existence of a closed loop originating from the PAG and finally returning to the PAG through the relays of NAcc (Ma et al., 1992). Yet, studies in this line are sparse. Our data demonstrates bi-directional modulation. dPAG priming modulated vSub HFS induced- plasticity in both the BLA and NAcc. Moreover, these modulatory effects were sensitive to the intensity of dPAG priming. While, under more “extreme” priming stimulation (1.0 mA) robust plasticity in the form of LTD was observed in both the BLA and NAcc, a more “moderate” stimulation (0.5 mA) resulted in differential effects; with an LTP in the BLA and a blockage of plasticity in the NAcc. These results are in line with previous findings that found dPAG stimulation intensity-dependent inhibition (e.g., Deakin and Graeff, 1991; Graeff et al., 1993). Unlike the classical autonomic role attributed to the PAG (Behbehani, 1995), Miranda-Paiva et al. (2003) suggested an integrative role for the PAG in influencing the selection of adaptive behavioral responses. The modulatory effects found in this study may hint to the ability of the PAG to induce a shift in the balance in higher limbic regions and to modulate the selection of adaptive responses. Further, it seems that the NAcc is more susceptible to the effects of priming of the limbic midbrain as was reflected by blockage of the vSub HFS –induced plasticity. Together with studies that indicated the NAcc as a central regulator for emotional behaviors (Covington et al., 2009; Vialou et al., 2010; Christoffel et al., 2011b), such an effect on NAcc plasticity could subsequently affect activity in other brain regions (such as cortical and limbic system regions) interacting with it (Mogenson et al., 1980). Overall, the effects of dPAG priming on plasticity in the BLA and the NAcc suggest a form of *metaplasticity* (Schmidt et al., 2013) that may be induced by the dPAG. dPAG HFS by itself failed to induce plasticity, but HFS to the vSub enabled these *metaplasticity* effects of dPAG priming to be revealed. When considering dPAG action in normal behaving animals, most studies focus on its immediate stimulation consequences. Studies in this line of research mainly deal with the PAG’s processing and modulation of pain, autonomic regulation, vocalization, and fear-related lordosis (Behbehani, 1995). Based on our findings, it seems that in addition, dPAG might have prolonged effects as well. In the current study we did not observe significant effects for dPAG stimulation by itself but did find profound intensity-dependent *metaplasticity* in the BLA and NAcc following dPAG stimulation, which may hint for its possible prolonged effects on other limbic regions. Therefore, it is important that future studies dealing with dPAG’s functional roles would focus not only on the PAG’s roles *per se* but also on its influence on activity and plasticity in other brain regions, such as the BLA and the NAcc. Recently, Kincheski et al. (2012) suggested that the dlPAG is specifically capable of interfering with emotional judgments and mnemonic processes by supporting fear learning to life threatening situations. An ample body of evidence focused on PAG’s important position in mediating the effects of predator stress induced-anxiety on a range of behaviors in rodents (Canteras and Goto, 1999; Adamec, 2001; Adamec et al., 2001, 2011; Cezario et al., 2008). We have recently investigated in rats, the functional relationship between the PAG and amygdala in a fear conditioning setting and in a naturalistic foraging setting (Kim et al., 2013). It was found that the dPAG is capable of conveying unconditioned stimulus information that can direct both innate and learned fear responses. It would be interesting to examine these effects also with more naturalistic settings such as those involving predatory stress since it has been suggested that the dPAG may be predominantly associated with response to such threats.

To summarize, we found that vSub stimulation simultaneously affects brain structures that are traditionally associated with either negative (BLA) or positive (NAcc) affect. This suggests that the traditional separation in studying these region’s functions may overlook an important aspect of their complementary roles. Examining the parallel plasticity in both structures enables examining the relative experience-induced alterations in these brain structures and by this help elucidating the integrative systems level plasticity associated with related altered behaviors. Furthermore, the current findings emphasize the potential contribution of midbrain structures, such as the dPAG to “higher-level” modulatory inputs from e.g., the mPFC (Marek et al., 2013; Richard and Berridge, 2013). One could envisage “two-arms” modulation on emotional responses, one involving a more “cognitive” modulation while the other a form of an “autonomic” modulation. Imbalance between these modulatory inputs may result in abnormal emotional responses, even in the absence of any impairment in the amygdala or NAcc *per se*.

## Conflict of interest statement

The authors declare that the research was conducted in the absence of any commercial or financial relationships that could be construed as a potential conflict of interest.
